# Identification and functional analysis of promoters of heat-shock genes from the fall armyworm, *Spodoptera frugiperda*

**DOI:** 10.1038/s41598-020-59197-8

**Published:** 2020-02-11

**Authors:** Xien Chen, Anjiang Tan, Subba Reddy Palli

**Affiliations:** 1 Department of Entomology, College of Agriculture, Food and Environment, University of Kentucky, Lexington, KY 40546 United States of America; 2 Key Laboratory of Insect Developmental and Evolutionary Biology, Center for Excellence in Molecular Plant Sciences, Shanghai Institute of Plant Physiology and Ecology, Chinese Academy of Sciences, Shanghai, 200032 China

**Keywords:** Zoology, Entomology

## Abstract

The functional information on heat-shock proteins (Hsp) and heat-shock promoters from an important agricultural insect pest, *Spodoptera frugiperda*, is still lacking. We conducted a genome-wide identification of *Hsp* genes and identified a total of 21 genes belonging to four major insect Hsp families (small heat-shock proteins, Hsp60, Hsp70, and Hsp90) in *S. frugiperda*. Expression of most of *S. frugiperda* (*SfHsp*) genes could be detected in Sf9 cells, embryos and larval tissues of *S. frugiperda*. The heat-inducible activity of heat-shock promoters from several *SfHsp* genes was tested in Sf9 cells and embryos. The promoter of *SfHsp70D* showed the high constitutive activity in cell line and embryos, while the activity of *SfHsp20.15* and *SfHsp20.71* promoters was most dramatically induced in Sf9 cells and embryos. In embryos, the heat-induced activity of *SfHsp20.71* and *SfHsp70D* promoters outperformed commercially used *ie1* and *ie2* promoters. The heat-induced activity of *SfHsp70D* and *SfHsp19.07* promoters were more robust than *ie2* promoter in Sf9 cells. These *SfHsp* promoters with high basal activity or with heat-induced activity from low basal activity, could be used in *S. frugiperda* or other lepidopteran insects for many applications including transgenesis and genome editing.

## Introduction

Heat-shock proteins (Hsp) are abundant and ubiquitously expressed in insects playing important roles in enhancing abiotic and biotic stress tolerance, as well as regulating normal development^1,2^. Based on their molecular mass and function, insect Hsp can be divided into four major families, small heat-shock proteins, Hsp60, Hsp70, and Hsp 90^2^. Expression of Hsp from a wide range of insect species have been reported to be induced and modulated by abiotic stressors, including extreme temperature^3–5^, ultraviolet radiation^6,7^, pesticides^8,9^, heavy metals^10,11^, desiccation^12–14^, starvation^15,16^, and anoxia/hypoxia^17–19^, as well as several biotic insults, including parasites^20,21^, pathogens^22,23^, and high population density^24^. In recent years, the availability of both transcriptome and genome data greatly contributed to the identification of an increasing number of *Hsp* genes from diverse insect species and promoted their functional studies^5,9,25–27^. However, information of *Hsp* genes from a destructive insect pest, *Spodoptera frugiperda*, is still limited^23,28^.

The stress-inducible expression of *Hsp* gene is conferred by binding of heat-shock factor (HSF) to heat-shock elements (HSEs), which consists of arrays of the 5-bp unit NGAAN arranged as inverted repeats in the promoter region^29^. Since exposure to high temperature is likely the simpler way to achieve inducible expression of insect *Hsp* genes, the promoters of insect *Hsp* genes are good candidates to drive the expression of foreign genes by heat-shock. Analysis of heat-shock promoters is still limited to a few model insect species. Promoters of *Hsp26*, *Hsp68*, *Hsp70*, and *Hsp82*, from *Drosophila melanogaster* have been used to drive the heat-shock induced expression of transposases facilitating the germline transformation in insects including those belong to order Coleoptera^30,31^, Diptera^32–36^, Hymenoptera^37^, and Lepidoptera^38–41^. The promoter of *D. melanogaster Hsp70* was also successfully used to establish a transient expression system for foreign protein production in Sf9 cells^42^. Two promoters of *Hsp*70 genes from *Aedes aegypti* showed robust heat-inducible activity in transgenic mosquitoes. Recently, promoter of *Hsp68* from *Tribolium castaneum* was used to improve germline transformation in this insect^43^. Identification and analysis of heat-shock promoters form other insect species could benefit the conditional expression of foreign genes in insects and cell lines developed from insects.

In this work, we identified multiple *Hsp* genes from *S. frugiperda*, and analyzed their heat-inducible expression in Sf9 cells, embryos, and different tissues of larvae. The potential promoters from several highly induced *Hsp* genes were cloned into the luciferase expression vector and evaluated their activity in Sf9 cells and embryos. Several promoters with activity in Sf9 cells and embryos were identified. The promoters with strong heat-inducible activity could be used for expression of proteins as well as for the development of transgenic and genome editing methods in this and other lepidopteran insects.

## Results

### *SfHsp* genes and their promoters

By blasting the Transcriptome Shotgun Assembly of *S. frugiperda* available at NCBI, orthologs of 21 heat-shock protein genes were identified and named as *SfHsp11.2*, *SfHsp15.82*, *SfHsp19.07*, *SfHsp19.35*, *SfHsp19.66*, *SfHsp19.74*, *SfHsp20.15*, *SfHsp20.71*, *SfHsp21.37*, *SfHsp21.38*, *SfHsp21.96*, *SfHsp24.35*, *SfHsp26.61*, *SfHsp29.00*, *SfHsp60, SfHsp70A*, *SfHsp70B*, *SfHsp70C*, *SfHsp70D*, *SfHsp75*, *SfHsp83*, and *SfHsp97* based on the predicted molecular weights of proteins encoded by these genes (The accession numbers of these genes are shown in Table S1). Phylogenetic analysis showed that heat-shock proteins are conserved among lepidopteran insects (Figs. S1 and S2).

Three types of HSEs, tail-tail, head-head, and step/gap^44^, were identified within the 2 kb-long potential promoter regions of 11 *SfHsp* genes (*SfHsp19.74*, *SfHsp20.15*, *SfHsp20.71*, *SfHsp21.37*, *SfHsp29.00*, *SfHsp70A*, *SfHsp70B*, *SfHsp70C*, *SfHsp70D*, *SfHsp83*, and *SfHsp97)*. No HSEs were found in the promoter region of *SfHsp19.66*. One or two types of HSEs were detected in the promoter regions of other *Hsp* genes. Maximum number of HSEs, 26 HSEs, were identified in the potential promoter of *SfHsp70D* (Table 1). Promoter sequence of SfHsp20.71 gene with potential HSE marked are shown in Fig. S3.

**Table 1:** Number of HSEs within 2 kb sequence upstream of ATG.

	Tail-tail	Head-head	Step/gap	Total
SfHsp11.2	0	2	4	6
SfHsp15.82	6	0	2	8
SfHsp19.07	0	8	4	12
SfHsp19.35	0	1	4	5
SfHsp19.66	0	0	0	0
SfHsp19.74	2	6	4	12
SfHsp20.15	4	4	3	11
SfHsp20.71	6	4	8	18
SfHsp21.37	6	6	5	17
SfHsp21.38	2	0	3	5
SfHsp21.96	0	0	4	4
SfHsp24.35	0	0	1	1
SfHsp26.61	0	4	0	4
SfHsp29.00	6	4	2	12
SfHsp60	2	4	3	9
SfHsp70A	6	6	3	15
SfHsp70B	4	4	3	11
SfHsp70C	4	4	4	12
SfHsp70D	8	10	8	26
SfHsp75	2	0	0	2
SfHsp83	8	8	3	19
SfHsp97	2	5	4	11

The sequences of 15 bp length HSEs are NTTCNNGAANNNNNN for Tail-tail type, NGAANNTCCNNNNNN for Head-head type, and NTTCNNNNNNNTTCN for Step/gap type. N is any nucleotide.

### Heat-shock induced expression of *SfHsp* genes

**Figure 1:**
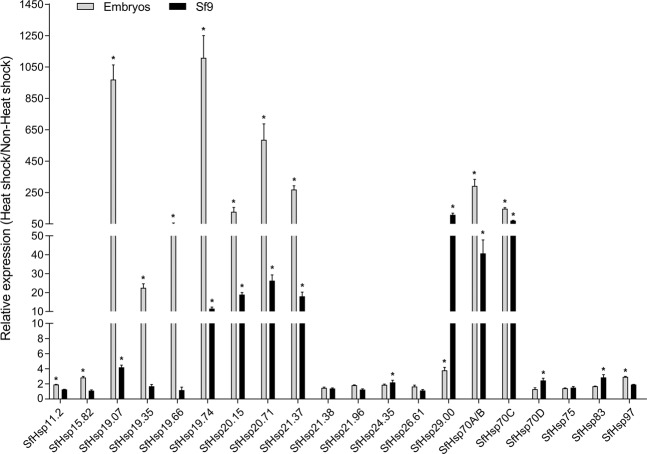
Heat-shock induced expression of *SfHsp* genes in Sf9 cells and embryos. The Sf9 cells and embryos were exposed to 37 °C for 1 hr, then let them recover at 27 °C for 1 hr. Cells and fresh embryos kept at 27 °C were used as non-heat-shock control. Total RNA was isolated, converted to cDNA and used in RT-qPCR to quantify mRNA levels. 28 s rRNA gene was used as the reference gene. Fold induction of heat-shock treatment over non-heat-shock control was calculated by the 2^−∆∆Ct^ method. Mean ± SD (n = 3) are shown. Data were analyzed using independent samples *t*-test built-in SPSS software. *Significantly different at *p* < 0.05.

Heat-shock response of *SfHsp* genes was first investigated in the embryos within 2 hr after oviposition and ovary-derived cell line, Sf9. Due to the presence of multiple melting peaks of *SfHsp60* amplification found in the melt curve analysis (data not shown), this gene was not included in the expression studies. The nucleotide sequences of *SfHsp70A* and *SfHsp70B* are highly similar. The same primers were used for the analysis of *SfHsp70A* and *SfHsp70B* expression. All *SfHsp* genes were expressed in embryos and Sf9 cells (Fig. S4), and most of the *SfHsp* genes were induced by heat-shock (Fig. 1). In embryos, expression of *SfHsp19.74* was up-regulated by 1,108.49-fold. Expression of *SfHsp19.07*, *SfHsp20.71*, *SfHsp70A/B*, *SfHsp21.37*, *SfHsp70C*, and *SfHsp20.15* were up-regulated by 970-, 585-, 291-, 269-, 146-, and 127-fold, respectively. The mRNA levels of *SfHsp19.35* was induced by 22-fold. Other *SfHsp* genes showed less than four-fold increase in their mRNA levels in heat-shocked embryos. In Sf9 cells, mRNA levels of *Sf29.00* increased the most, by 108-fold after heat-shock. The mRNA levels of *SfHsp19.74, SfHsp20.15, SfHsp20.71, SfHsp21*.37, *SfHsp70A/B*, and *SfHsp70C* were increased by 11.52- to 71.07-fold, respectively (Fig. 1). It appears that the heat-shock response of *SfHsp* genes in embryos is more pronounced than in the cell line.

**Figure 2:**
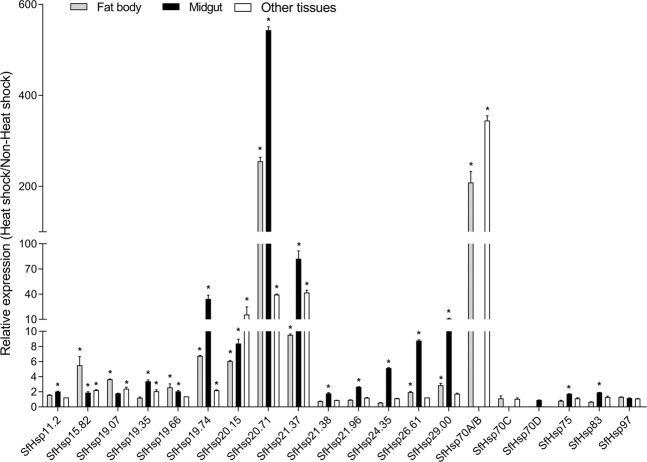
Heat-shock induced expression of *SfHsp* genes in larval tissues. The 6^th^ instar larvae were exposed to 37 °C for 1 hr, then let them recover at 27 °C for 1 hr. Fat body, midgut, and the rest of the tissues were dissected and used for quantifying mRNA levels as described in Fig. 1 legend.

Expression of *SfHsp* genes after heat-shock was also determined in the midgut, fat body, and other tissues from 6^th^ instar larvae. The mRNA of *SfHsp70A/B* and *SfHsp70C* were not detected in the midgut, while *SfHsp70D* was expressed only in the midgut (Fig. S5). All other *SfHsp* genes were expressed in all tested tissues. Most of the *SfHsp* genes showed heat-shock response in different tissues tested (Fig. 2). In the fat body, expression levels of *SfHsp20.71* and *SfHsp70A/B* were induced by 255.08- and 208.46-fold, respectively. Expression of *SfHsp15.82, SfHsp19.74*, *SfHsp20.15*, and *SfHsp21.37* were up-regulated by 5.51-, 6.74, 6.05-, and 9.52-fold, respectively. Other *SfHsp* genes showed less than 4-fold heat-shock induction. In the midgut, *SfHsp20.71* mRNA displayed the most remarkable increase of 543-fold, and expression of *SfHsp21.37* was also enhanced by 82-fold. Expression of *SfHsp19.74, SfHsp20.15, SfHsp24.35, SfHsp26.61*, and *SfHsp29.00* increased by 5- to 34-fold. In other mixed tissues, only four SfHsp genes were induced by heat-shock (*SfHsp70A/B* at 344-fold, *SfHsp20.15* at 16-fold*, SfHsp20.71* at 40-fold, and *SfHsp21*.37 at 42-fold).

### Analysis of promoter activity

Based on the heat-shock response of *SfHsp* genes, potential promoters of seven highly induced genes (*SfHsp19*.07, *SfHsp19.74*, *SfHsp20.15*, *SfHsp20.71*, *SfHsp21.37*, *SfHsp29.00*, and *SfHsp70A)* were chosen to conduct the promoter activity test. The potential promoter of *SfHsp70D*, containing the maximum number of HSEs, was also included in the promoter activity test. The nucleotide sequences upstream to the ATG, harboring most of the HSEs in the potential promoters, were amplified, yielding 790 bp (14 HSEs) for *SfHsp19.07*, 1385 bp (12 HSEs) for *SfHsp19.74*, 872 bp (7 HSEs) for *SfHsp20.15*, 1638 bp (18 HSEs) for *SfHsp20.71*, 1369 bp (16 HSEs) for *SfHsp21.37*, 499 bp (11 HSEs) for *SfHsp29.00*, 1218 bp (14 HSEs) for *SfHsp70A*, and 1403 bp (25 HSEs) for *SfHsp70D* fragments. These fragments were cloned into pGL5luc vector to obtain *SfHsp*-promoter-pGL5luc plasmids.

**Figure 3:**
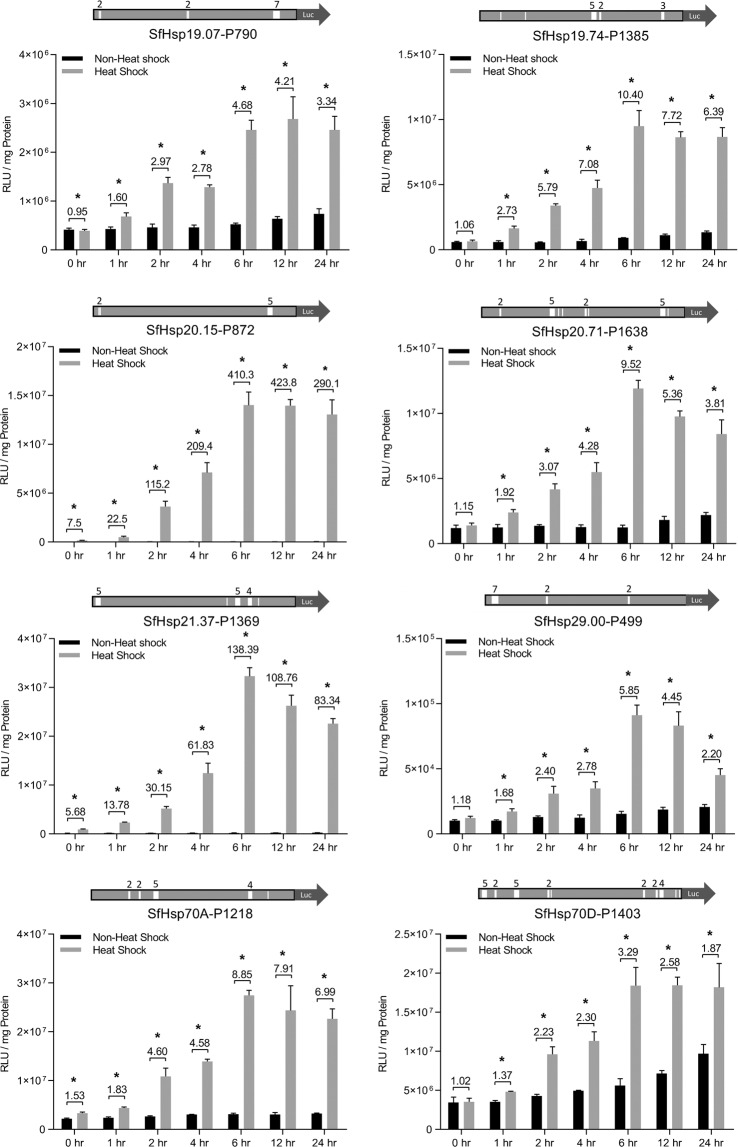
Time-course assay of *S. frugiperda* heat-shock promoters in Sf9 cells. Structure of each heat-shock promoter construct is shown on the top of each panel, with a solid rectangle of light gray representing heat-shock promoter, and solid arrow of dark gray indicating the open reading frame of firefly luciferase. The approximate locations of HSEs are indicated with white vertical bars, with the numbers above representing the number of HSEs in each HSEs cluster. 100 ng of heat-shock promoter construct or pGL5luc vector was transfected into Sf9 cells. At 48 hr post-transfection, the cells were exposed to 37 °C for 1 hr, followed by recovery at 27 °C for 0, 1, 2, 4, 6, 12, and 24 hr. The cells were harvested, lysed, and the luciferase activity and protein concentration were determined. Transfected cells kept at 27 °C were used as non-heat-shock control. Numbers on the top indicate the increase in fold induction after heat-shock. Mean ± SD (n = 5) are shown. Data were analyzed using independent samples *t*-test built in SPSS software. *Significantly different at *p* < 0.05.

In Sf9 cells, a time-course measurement of the luciferase activity was carried out at 0, 1, 2, 4, 6 12, and 24 hr post-heat-shock. All the eight constructs supported an increase in the luciferase activity from 0 hr to 6 hr post-heat-shock (Fig. 3). The maximum activity was maintained at 12 and 24 hr after heat-shock (Fig. 3). The rank of basal activities of eight promoters is *SfHsp70D*-P1403 > *SfHsp70A*-P1218 > *SfHsp20.71*-P1638 > *SfHsp19.74*-P1385 > *SfHsp19.07*-P790 > *SfHsp21.37*-P1369 > *SfHsp20.15*-P872 > *SfHsp29.00*-P499. The rank of heat-shock induced activities of eight promoters at 6 hr post heat-shock is *SfHsp21.37*-P1369 > *SfHsp70A*-P1218 > *SfHsp70D*-P1403 > *SfHsp20.15*-P872 > *SfHsp20.71*-P1638 > *SfHsp19.74*-P1385 > *SfHsp19.07*-P790 > *SfHsp29.00*-P499. At 6 hr post-heat-shock, *SfHsp20.15*-P872-pGL5luc and *SfHsp21.37*-P1369-pGL5luc constructs showed 410- and 138-fold heat-induced enhanced of the luciferase activity, respectively. The heat-induced luciferase activity of the other six constructs increased by 3- to 10-fold (Fig. 3).

**Figure 4:**
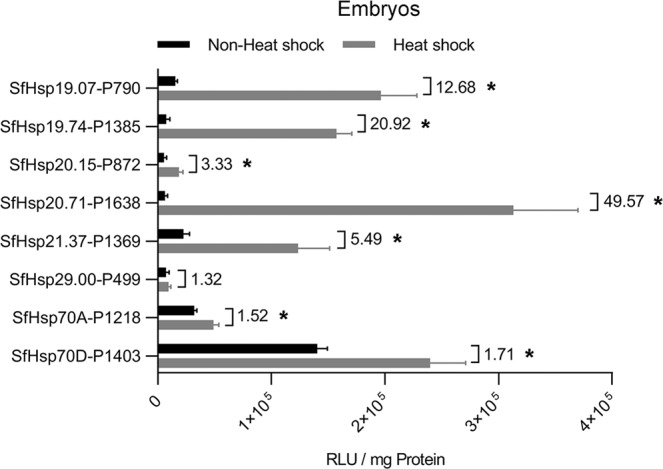
Promoter activity assay in embryos. A mixture containing 1.0 μg/μL of promoter construct or pGL5luc vector and 0.5 μg/μL of pBac-hr5/*ie1*-EGFP-SV40 plasmid was injected into eggs within 2 hr after oviposition. At 24 hr post-injection, the eggs were exposed to 37 °C for 1 hr, then kept at 27 °C for 6 hr. The EGFP expressing eggs were collected, and the luciferase activity and protein concentration were determined. Injected eggs kept at 27 °C were used as non-heat-shock control. Numbers on the top indicate the fold induction after heat-shock. Mean ± SD (n = 5, Sf9; n = 3, embryos) are shown. Data were analyzed using independent samples *t*-test built-in SPSS software. *Significantly different at *p* < 0.05.

To examine the luciferase activity of SfHsp-promoter-pGL5luc constructs in embryos, the constructs were injected into embryos along with an EGFP expression vector. In preliminary experiments, we found that, at 24 hr post-injection, only the living fertile embryos showed the visible transient expression of EGFP, which facilitated the selection of embryos for luciferase activity test. The presence of EGFP had no effect on luciferase activity. Unlike in cell lines, promoter of *SfHsp20.15*-P872 showed only 3.3-fold heat-inducible activity in embryos (Fig. 4). *SfHsp20.71*-P1638-pGL5luc construct showed the highest (50-fold increase) luciferase activity after heat-shock. Promoters of *SfHsp19.74*-P1385 and *SfHsp19.07*-P790 also displayed strong heat-inducible activity at 20.92- and 12.68-fold increase, respectively. The luciferase activity of the other four constructs was increased by 1.32- to 5.49-fold (Fig. 4). Similar relativity activity of these promoters was observed when the luciferase activity was normalized with EGFP activity from a co-transfected construct (Fig. S6). These data showed that the promoter of *SfHsp20.71*-P1638 could be used to drive controlled expression of endogenous or exogenous genes in embryos.

**Figure 5:**
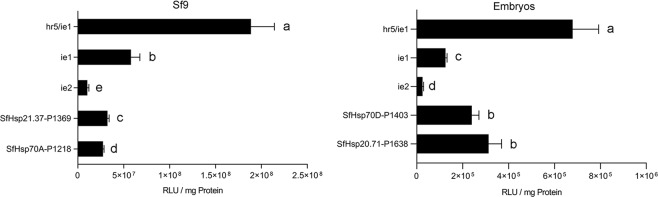
Comparison of highly heat-inducible *S. frugiperda* heat-shock promoters with commercially used promoters. The top two heat-shock promoter constructs that showed heat-inducible activity in Sf9 cells or embryos and *ie1*, *ie2*, and hr5/*ie1* constructs were transfected into Sf9 cells or injected into embryos. The transfected cells and injected eggs were processed as described in Fig. 4 legend. Mean ± SD (n = 5) are shown. Different letters beside each column indicate significant differences (at *p* < 0.05) among multiple groups, which were determined using one-way ANOVA followed by the Tukey HSD test.

Four constructs with the highest heat-inducible activity, *SfHsp21.37*-P1369-pGL5luc, and *SfHsp70A*-P1218-pGL5luc in Sf9 cells and *SfHsp70D*-P1403-pGL5luc and *SfHsp20.71*-P1638-pGL5luc in embryos were selected for comparing their performance with the promoters (*ie1*, hr5/*ie1*, and *ie2*) currently used for expression of genes in insect cells. These constructs along with hr5/*ie1*-pGL5luc, *ie1*-pGL5luc, and *ie2*-pGL5luc were transfected into cells or injected into embryos. The luciferase activity was measured at 6 hr post-heat-shock as described above. All *SfHsp* promoter constructs tested showed higher activity than *ie2* construct. However, they displayed lower activity than hr5/*ie1* construct in cell line and embryos. The luciferase activity of both *SfHsp*-promoter constructs was lower than that of *ie1* construct in Sf9 cells and embryos (Fig. 5).

## Discussion

Since the increase in synthesis of heat-shock proteins (Hsp) after heat-shock were reported in *D. melanogaster*^45^, a large number of *Hsp* genes have been identified in many insect species as important modulators of survival under environmental stresses^1^, as well as crucial regulators of normal development and diapause^2^. Inducible expression of insect *Hsp* genes has been extensively studied; however, functional information on heat-shock promoters is still lacking. In this study, genome-wide identification of *S. frugiperda Hsp* genes, as well as functional analysis of heat-inducible promoters were conducted.

Similar to the findings in other insects^5,25,27^, the expression of several *Hsp* genes was induced by heat-shock in *S. frugiperda* cell line, embryos, and larval tissues. Since heat-inducible expression of *Hsp* gene was achieved by HSF binding to the HSEs located in the promoters of *Hsp* genes^29^, the variation of heat-inducibility among *SfHsp* gene is likely associated with the number of HSEs in their promoters. We found that most *SfHsp* genes possessing more than 10 HSEs in their promoters were significantly up-regulated in at least one tested system after heat-shock at 37 °C. However, to our surprise, *SfHsp83* containing 19 HSEs displayed quite low heat-inducibility in cell line and embryos (Table 1). The previous study found that the induction of the *SfHsp90* (*SfHsp83* in this study) occurred at 42 °C, but not at 37 °C^28^. Expression of *SfHsp83* might be induced at a higher temperature. It is interesting that induction of non-HSE containing *SfHsp19.66* was detected in embryos. These data suggest that the heat-shock induction of *SfHsp* genes may depend not only on temperature and number of HSEs, but also on other factors yet to be identified. Promoters of *ie1* and *ie2* are most commonly used in transient gene expression studies in insect cells^46–50^. However, lower expression of foreign proteins in insect cells was observed when using *ie1*/*ie2*-based transient gene expression systems^51–53^. Recently, a *Drosophila Hsp70* promoter based transient gene expression system was established to produce foreign proteins by heat-shock in Sf9 cells^42^. These heat-inducible *SfHsp* promoters could be used for establishing novel heat-induced transient gene expression system in lepidopteran cell lines.

In early embryos, promoters of *SfHsp70* and *SfHsp20.71*, showed the highest basal activity and heat-inducible activity, respectively (Fig. 4). These promoters are more active than the commercially used *ie2* promoter (Fig. 5). In insect transgenic studies, helper plasmids containing promoters of *Drosophila* Hsp genes have been widely used for successful germline transformation in many insect species^54^. However, there is no report about using endogenous heat-shock promoters for germline transformation in lepidopteran insects, which is mainly due to lack of functional information on lepidopteran heat-shock promoters. Promoters of *SfHsp70* and *SfHsp20.71* could be used for driving expression of transposases in germline transformation of *S. frugiperda*.

## Conclusion

In conclusion, we identified and characterized multiple *Hsp* genes from an important lepidopteran pest, *S. frugiperda*. The heat-inducible activity of several *SfHsp* promoters was also analyzed. We identified several promoters with strong heat-inducible activity, which could be used for protein production, as well as for development of transgenic and genome editing methods in this insect. Because of conservation among lepidopteran *Hsp* genes, promoters of *SfHsp* genes could also function in other lepidopteran insects and therefore could be used to generate transgenic insects.

## Methods

### Insect and cells

The laboratory strain of *S. frugiperda* was purchased from Benzon Research Inc. (Pennsylvania, USA). Adults were fed with 10% sucrose solution. The eggs laid on paper towel were collected, and larvae were reared on artificial diet purchased from Southland Product Inc. (Arkansas, USA). Sf9 cells were maintained at 27 °C in Sf-900 II medium (Thermo Fisher, USA).

### Identification and analysis of *SfHsp* genes

The putative *SfHsp* genes were identified from Transcriptome Shotgun Assembly of *S. frugiperda* available from NCBI using nucleotide sequences of *S. litura Hsp* genes as queries. Their deduced amino acid sequences were subjected to the non-redundant database on NCBI to confirm homology with other insect Hsp proteins. To analyze the relationship of small *Hsp* genes among lepidopteran insects, a phylogenetic tree was constructed based on the amino acid sequences of small *Hsp* genes from *B. mori*, *Danaua plexippus*, *P. xylostella*, *S. frugiperda*, and *S. litura*. Another phylogenetic tree was also constructed based on the amino acid sequences of Hsp60, Hsp70, Hsp75, Hsp83, and Hsp97 from *B. mori*, *D. melanogaster*, *T. castaneum*, *S. frugiperda*, and *S. litura*. Phylogenetic trees were obtained by MEGA7^55^ using the neighbor-joining method with a bootstrap test of 1,000 replicates.

The putative promoter sequences were obtained from Whole-genome shotgun contigs of *S. frugiperda* available from NCBI using nucleotide sequences of identified *SfHsp* genes as queries. The consensus heat-shock elements (HSEs) present in the 2 kb putative promoter region upstream to the ATG site were identified as described previously^44^.

### Heat-shock assays of *SfHsp* genes

Eggs, Sf9 cells and 6^th^ instar larvae were exposed to 37 °C for 1 hour, then recovered at 27 °C for 1 hr. Cells were directly subjected to RNA extraction using TRI reagent (Molecular Research Center Inc., USA). Larval tissues including midgut, fat body, and the remaining tissues were dissected for RNA preparation. Cells and animals maintained at 27 °C were used as non-heat-shock controls. Each treatment included three biological replicates. The tissues and cells were stored in −80 °C until RNA extraction. Total RNA was extracted using TRisol reagent (MRC laboratories, Cincinnati, OH). Complementary DNAs (cDNAs) were synthesized from 1.0 μg total RNA using the M-MLV reverse transcriptase kit (Invitrogen, USA) and stored at −20 °C. Using 20-fold diluted cDNAs as templates, real-time PCR reactions were conducted in a 10-μL total reaction volume containing 5 μL of 2xSYBR Mixture (BioRad, USA), 0.4 μL of each primer, 0.8 μL of cDNA, and 3.2 μL of double-distilled water. The reaction conditions were as follows: 95 °C for 2 min, 40 cycles of 95 °C for 10 s, and 60 °C for 1 min, then followed by a dissociation analysis. For each gene, the reactions included three technical replicates. Basal expression levels of *SfHsp* genes were represented as fold change over the expression levels of reference gene 28 s rRNA. Fold induction were calculated with the 2^−∆∆Ct^ method^56^ between treatment and control samples for each biological replicate. Primers were generated by online tool, Primer3 (http://bioinfo.ut.ee/primer3-0.4.0/), and listed in Table S2.

### *SfHsp* promoter-reporter constructs

Sequences containing most of the HSEs in putative promoters of *SfHsp19.07*, *SfHsp19.74*, *SfHsp20.15*, *SfHsp20.71*, *SfHsp21.37*, *SfHsp29.00*, *SfHsp70A*, and *SfHsp70D*, were amplified from genomic DNA using Prime STAR GXL DNA Polymerase (TakaRa, Japan). Promoter sequences of *ie1* (immediate-early gene 1 of AcMNPV) and hr5/*ie1* were amplified using pBac-hr5/*ie1*-EGFP-SV40 plasmid as a template, and promoter sequence of *ie2* (immediate-early gene 2 of OpMNPV) was amplified from pIZT/V5-His vector (Invitrogen, USA). All amplified products were cloned into pGL5luc upstream of the firefly luciferase open reading frame, yielding plasmids of *SfHsp19.07*-P790-pGL5luc, *SfHsp19.74*-P1385-pGL5luc, *SfHsp20.15*-P872-pGL5luc, *SfHsp20.71*-P1638-pGL5luc, *SfHsp21.37*-P1369-pGL5luc, *SfHsp19.00*-P499-pGL5luc, *SfHsp70A*-P1218-pGL5luc, *SfHsp70D*-P1403-pGL5luc, hr5/*ie1*-pGL5luc, *ie1*-pGL5luc, and *ie2*-pGL5luc. Primers used in the preparation of these constructs are listed in Table S2.

### Reporter assays

The cells were seeded into 96-well culture plates at a density of 2 × 10^5^ cells per ml and incubated at 27 °C overnight for transfection. Sf9 cells were transfected with 100 ng of promoter construct or pGL5luc vector per well using 0.8 μL of Cellfectin II reagent (Thermo Fisher, USA) in 50 μL of Sf-900 II medium (Thermo Fisher, USA). Four hours post-transfection, the medium was removed and replaced with 100 μL of fresh medium. At 48 hours post-transfection, the cells were exposed to 37 °C for 1 hr. The cells were recovered at 27 °C for 0, 1, 2, 4, 6, 12, and 24 hr, then harvested for luciferase activity assay. The medium was removed, and cells were washed with 100 μL of 1xPBS, then 100 μL of ice-cold lysis buffer was added into each well. The plates were placed on a shaker for 20 min at room temperature. 20 μL and 10 μL of cell lysate were used for the luciferase activity assay and protein concentration assay, respectively, as described^57^. Five replicates for each construct were performed.

Eggs were collected within 2 hr after oviposition and aligned on glass slides. A mixture containing 1.0 μg/μL of promoter construct or pGL5luc vector and 0.5 μg/μL of pBac-hr5/*ie1*-EGFP-SV40 plasmid was injected into aligned eggs. At 24 hr post-injection, eggs were exposed to 37 °C for 1 hr, then kept at 27 °C for 6 hr. The EGFP expressing eggs were collected and randomly divided into 3 groups with 20–30 eggs in each group. The pooled eggs in each group were homogenized with 200 μL of ice-cold lysis buffer, then centrifuged at 15,000xg for 30 min at 4 °C. 20 μL and 10 μL of supernatant extract were used for the luciferase activity assay and protein concentration determination respectively, as described^57^.

### Statistical analysis

For statistical analysis, IBM SPSS Statistic 25 was used. All data were shown as mean ± SD (standard deviation). The significant difference between two groups was analyzed using independent samples *t*-test; *p* < 0.05 was considered statistically significant. Significant differences among multiple groups were analyzed using one-way ANOVA followed by the Tukey HSD test.

## Supplementary information

Supplementary Information.

## Data Availability

All data generated or analyzed during this study are included in this published article and in additional information files.
