# Supplementary material for: Identification and functional analysis of promoters of heat-shock genes from the fall armyworm, Spodoptera frugiperda
Source: Sci Rep. 2020 Feb 11;10:2363. doi: 10.1038/s41598-020-59197-8 (PMC7012861; doi:10.1038/s41598-020-59197-8)
Supplement: Supplementary file 1 — Supplementary Information. [file 41598_2020_59197_MOESM1_ESM.pdf]

Identification and functional analysis of heat-shock promoters from *Spodoptera frugiperda*

Xien Chen [a], Anjiang Tan [b], Subba Reddy Palli [a, *]

[a] *Department of Entomology, College of Agriculture, Food and Environment, University of Kentucky, Lexington, KY 40546, United States of America*

[b] *Key Laboratory of Insect Developmental and Evolutionary Biology, Center for Excellence in Molecular Plant Sciences, Shanghai Institute of Plant Physiology and Ecology, Chinese Academy of Sciences, Shanghai, 200032, China.*

* Corresponding author. E-mail address: rpalli@uky.edu

Table S1 Accession of *SfHsp* sequences on NCBI.

Sequence	Accession number
SfHsp11.2	MN735761
SfHsp15.82	MN735762
SfHsp19.07	MN735763
SfHsp19.35	MN735764
SfHsp19.66	MN735765
SfHsp19.74	MN735766
SfHsp20.15	MN735767
SfHsp20.71	MN735768
SfHsp21.37	MN735769
SfHsp21.38	MN735770
SfHsp21.96	MN735771
SfHsp24.35	MN735772
SfHsp26.61	MN735773
SfHsp29.00	MN735774
SfHsp60	MN735775
SfHsp70A	MN735776
SfHsp70B	MN735777
SfHsp70C	MN735778
SfHsp70D	MN735779
SfHsp75	MN735780
SfHsp83	MN735781
SfHsp97	MN735782
SfHsp19.07-P790	MN735783
SfHsp19.74-P1385	MN735784
SfHsp20.15-P872	MN735785
SfHsp20.71-P1638	MN735786
SfHsp21.37-P1369	MN735787
SfHsp29.00-P499	MN735788
SfHsp70A-P1218	MN735789
SfHsp70D-P1403	MN735790

Table S2 Primers used in this study.

Primers	Sequence (5'-3')	Purpose
SfHsp11.2-QF	AGGGCTGAAGCAGTAACCAA	qPCR
SfHsp11.2-QR	TCGCTCTCTCGGAATAGGTG	qPCR
SfHsp15.82-QF	GTGCACGACTATGCTCCTGA	qPCR
SfHsp15.82-QR	TGCGAGGAACAGAGATGATG	qPCR
SfHsp19.07-QF	CCCGTTCTTCAGAGATCCAG	qPCR
SfHsp19.07-QR	ACCGTGATCTCGTTTCCAAC	qPCR
SfHsp19.35-QF	GGACCAGAACTTCGGACTGA	qPCR
SfHsp19.35-QR	TGGCCTTCAACCACAATGTA	qPCR
SfHsp19.66-QF	TTGCCTGAAGGGTGTATTCC	qPCR
SfHsp19.66-QR	ACTTCGGTTTTCGTGGTTTG	qPCR
SfHsp19.74-QF	GGGTATATTTCCCGCCAGTT	qPCR
SfHsp19.74-QR	CTGGTCCTTGACTTCCTTGC	qPCR
SfHsp20.15-QF	CCGCAAGACCAGTTAACCAT	qPCR
SfHsp20.15-QR	GATGAATCCGTGCTGATCCT	qPCR
SfHsp20.71-QF	AGCCAAGCATGAGGAGAAGA	qPCR
SfHsp20.71-QR	CTGGTCCTTGATCTCCTTGC	qPCR
SfHsp21.37-QF	CCTTTTGTTCTCGGCTACGA	qPCR
SfHsp21.37-QR	ATCCTTGTCGGACTTGATGC	qPCR
SfHsp21.38-QF	CGACACTGAATTCTCCAGCA	qPCR
SfHsp21.38-QR	CACTGTGCTGTGACGATGTG	qPCR
SfHsp21.96-QF	CGAGGAGAAACAGGATGAGC	qPCR
SfHsp21.96-QR	CTGTCTTGGTGATGGGGACT	qPCR
SfHsp24.35-QF	GCTGACATTGGACCCAGAAT	qPCR
SfHsp24.35-QR	CTCGAGGAGTTGGCTGGTAG	qPCR
SfHsp26.61-QF	GGTGTTCGACAAGGGAGTGT	qPCR
SfHsp26.61-QR	TGGCTTTGTCTTGGTCTCCT	qPCR
SfHsp29.00-QF	GAGGAAGTCCTGGAGGAACC	qPCR
SfHsp29.00-QR	GGTCACTTCTTCCTGCTTGC	qPCR
SfHsp75-QF	AGACAGACAGGCCTCTGGAA	qPCR
SfHsp75-QR	TGCCCTGCTTTTTGATCTCT	qPCR
SfHsp83-QF	GAAGCGCAAGAACAACATCA	qPCR
SfHsp83-QR	TTCTGTTGGAGCATCTCACG	qPCR
SfHsp97-QF	TGAGAACAGAAGGCGAACCT	qPCR
SfHsp97-QR	GCCTCTGCCACTTTCTGAAC	qPCR
SfHsp70A/B-QF	GTGGCTATGGAGGAAACCAG	qPCR
SfHsp70A/B-QR	CGCACATTTTCCTCTGTTGA	qPCR
SfHsp70C-QF	GGCCAACTGTGGAAGAAGTC	qPCR
SfHsp70C-QR	TGTGGGTGTGTTAGCGTTTG	qPCR
SfHsp70D-QF	CAAGCAAGCGAAGAGAAGAGA	qPCR

SfHsp70D-QR	CACACGAGTAGGTCGTTCCA	qPCR
SfHSP70A-P1218F	ATCGGGGTACCCACCTACACAC AGTTCGAATATTGC	Promoter amplification
SfHSP70A-P1218R	CCATGCCATGGTTTGTTTGTCTT CAGATTCTCTTCAC	Promoter amplification
SfHSP70D-P1403F	ATCGGGGTACCAACTCAATAAC CGGTTGGGTAAGC	Promoter amplification
SfHSP70D-P1403R	CCATGCCATGGTTTTCTTGATTA TATTTCTTCTTTATTCAC	Promoter amplification
SfHSP19.07-P790F	ATCGGGGTACCAGTTAATAGTCA CCTACACTCGGAAG	Promoter amplification
SfHSP19.07-P790R	CCATGCCATGGTTTGGTCGTTTTG TTTATTTCGTTTG	Promoter amplification
SfHSP19.74-P1385F	ATCGGGGTACCTTCTGCATAGTCT ATGGATATCGG	Promoter amplification
SfHSP19.74-P1385R	CCATGCCATGGTTTTTCTTATTTCG TTTTCAACTGAC	Promoter amplification
SfHSP20.15-P872F	ATCGGGGTACCTACTGGATGTATCT AGAGTGCTCC	Promoter amplification
SfHSP20.15-P872R	CCATGCCATGGCTTCACTGGATAAT ACTGATAATC	Promoter amplification
SfHSP20.71-P1638F	ATCGGGGTACCAACACTGACATGAA GACACATGG	Promoter amplification
SfHSP20.71-P1638R	ATCCCAAGCTTTTTGCTTGATTTCTAA CACAACAAC	Promoter amplification
SfHSP21.37-P1369F	ATCGGGGTACCACATACATTTCAAAG TAAGTCACG	Promoter amplification
SfHSP21.37-P1369R	CCATGCCATGGTTTGTTGAGTGTTGA GTATTCTTC	Promoter amplification
SfHSP29.00-P499F	ATCGGGTACCGAGTAGAGTAACCTCT ACTCTCC	Promoter amplification
SfHSP29.00-P499R	CCATGCCATGGCGTTCGTTGCG TTGTTGTTGCTTTC	Promoter amplification
ie1-F	ATCGGGGTACCCGATGTCTTTG TGATGCGCGCGAC	Promoter amplification
ie1-R	CCATGCCATGGCTTGGTTGTTCA CGATCTTGTCG	Promoter amplification
ie2-F	ATCGGGGTACCCATGATGATAAA CAATGTATGG	Promoter amplification
ie2-R	CCATGCCATGGAACAGATGCTGT TCAACTGTGT	Promoter amplification
hr5/ie1-F	ATCGGGGTACCCATTGCTTGTCAT TTATTAATTTGG	Promoter amplification
hr5/ie1-R	CCATGCCATGGCTTGGTTGTTCAC GATCTTGTCG	Promoter amplification

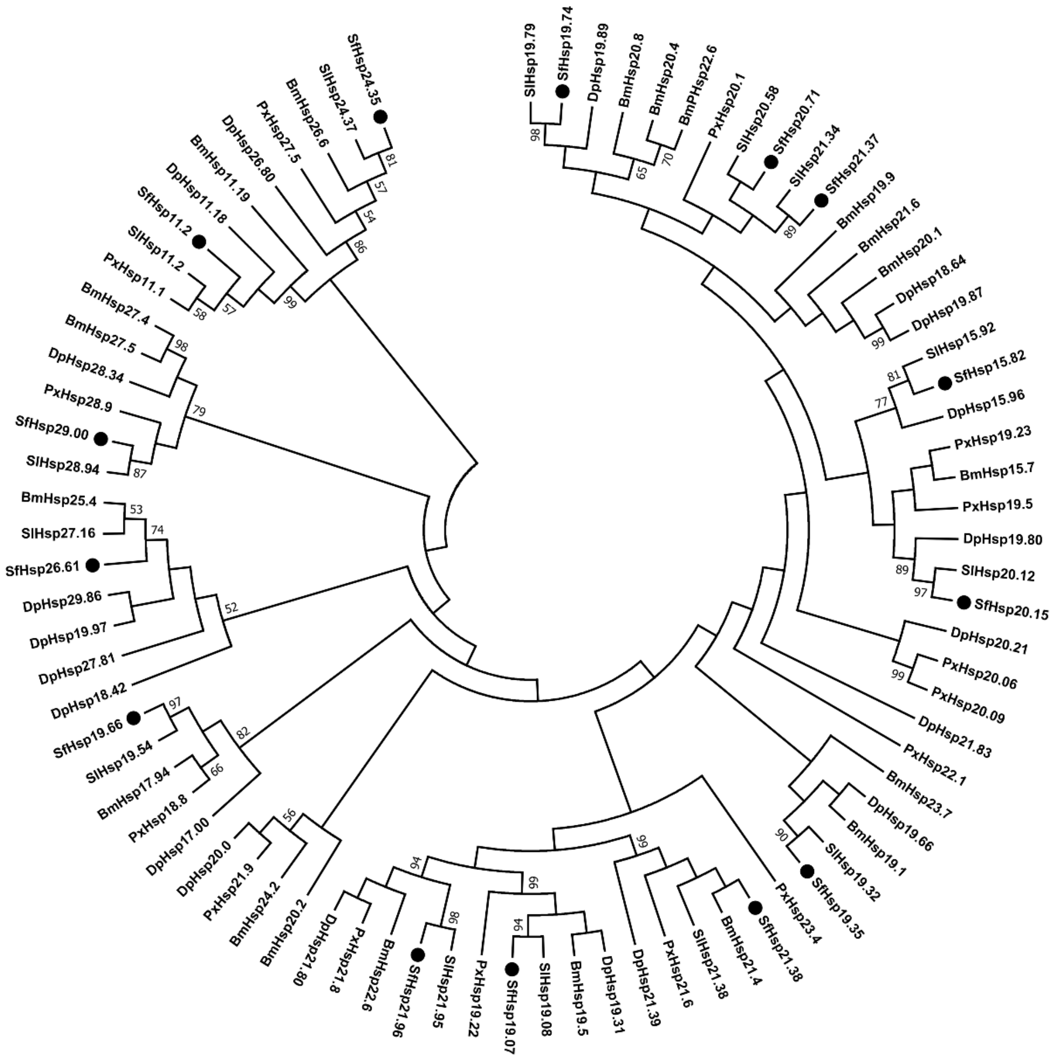

Figure S1. Phylogenetic analysis of lepidopteran small Hsp genes. Amino acid sequences of small Hsp of *Bombyx mori* (Bm), *Danaua plexippus* (Dp), *P. xylostella* (Px), *S. frugiperda* (Sf), and *S. litura* (Sl) were obtained from the non-redundant database on NCBI. Phylogenetic tree was established using MEGA7 (https://www.megasoftware.net) by neighbor-joining method with a bootstrap test of 1,000 replicates. Only percentage bootstrap values over 50 are indicated on the nodes of the tree. The small *S. frugiperda* Hsp are labeled with black dots.

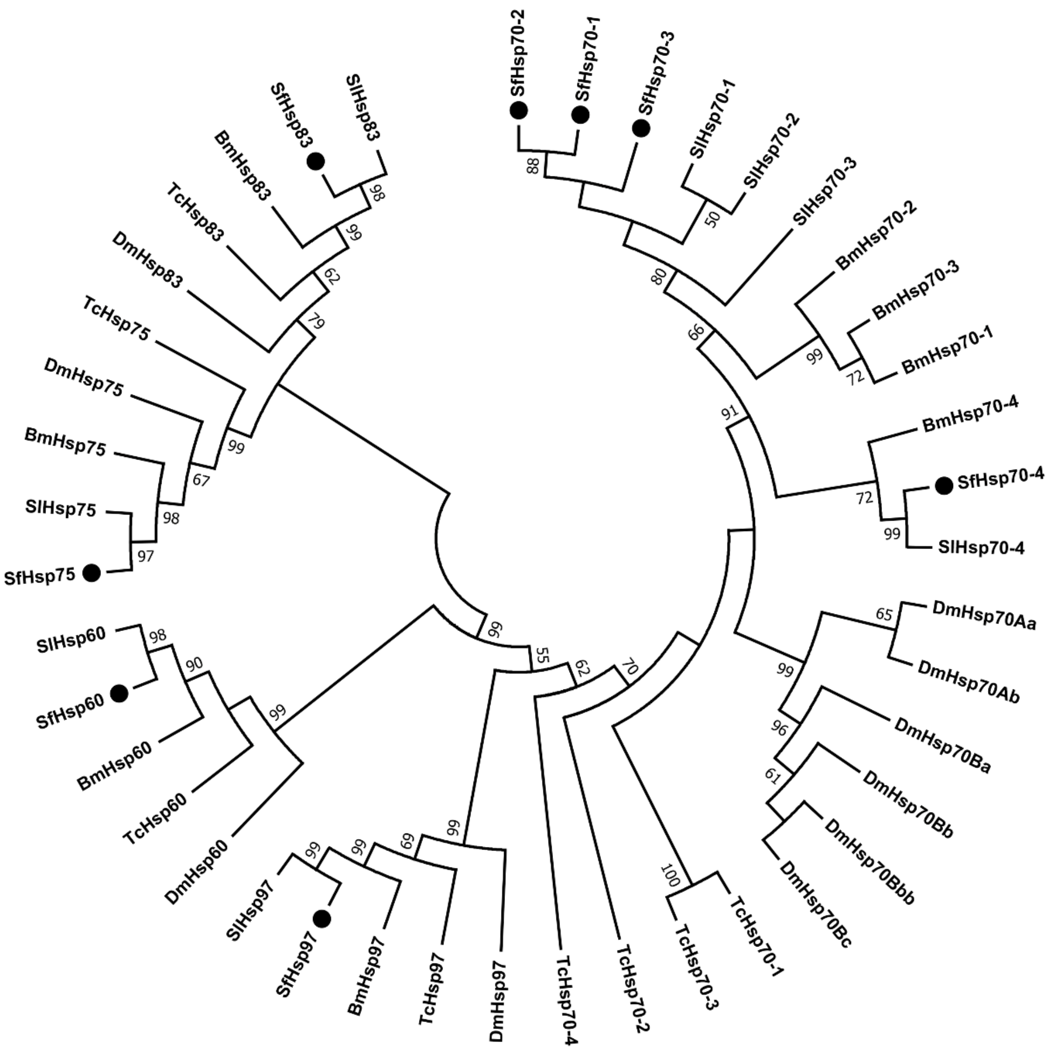

Figure S2. Phylogenetic analysis of insect Hsp60, Hsp70, Hsp75, Hsp83, and Hsp90. Amino acid sequences of Hsp60, Hsp70, Hsp75, Hsp83, and Hsp90 from *B. mori* (Bm), *Danaua plexippus* (Dp), *P. xylostella* (Px), *S. frugiperda* (Sf), and *S. litura* (Sl) were obtained from the non-redundant database on NCBI. The phylogenetic tree was established using MEGA7 (https://www.megasoftware.net) by the neighbor-joining method with a bootstrap test of 1,000 replicates. Only percentage bootstrap values over 50 are indicated on the nodes of the tree. The *S. frugiperda* Hsp are labeled with black dots.

CTGGATATGGCAATCGAGACGTCTAGCCAGTAAAAGATAAACCTAATGGGTATTTTTTTGGTATAAGCTAGTGAACGAGCAGGCAATTTTTT
TTTGTAAATGTGTAAACTAAACACTGACATGAAGACACATGGTAAACATATTATTTTTCCAGAAATAACTAACAGCTGATAGTGATTCAGCT
ACAAATCGTATTGTTTACAATGTAGGTTAGTAGATAAAGGTGATAATTGGTATTACCCGTCAGTTAAATCATCAAGAAATATTTATATCGCTA
GGTATTTTTTAATGTTTATTGATAAAAATAAACAAATAATATAAGTAAGTATTTTAATTAAGACTAAACCATATTCAAATTATTATTAAACATTA
CCTACCAAAACTAACGCAATAACAAAACGCAAGTCATCATATACATCATTGTCATAAAAATAATAATTATGTAACTCCAAAACGAATTCAAA
TCGTGATCGTGAACATTTCCAAACAATTCTAGAAAAGAAAATTCGATTCCACTTTCAAATTCAAATCGTAAACATTTCCGAACAATTCTATAA
AAGAAAAAAAAAATAAGCGACCATTCCATTTTCGAACATATTCTAATTTCTTTCAGTGTTAATTTAAATTGTATAATTTTGGCGCCAATTGCTCA
TGGCAACCATATTTTTATTATTAAATGATTATTGTCGCAATTTTATAATCAGTTTGTCCTTGGACATGACCTATACACAATCCTACTAGGTCTC
ATTAATTCAGTAGCATAGAACGTTGTAGAACGCCATGGAATGTTTTGTTCGCGAGGGAGTTGCCAGTCGATATTCTCGGCGGTTCCTCGGC
CGTTAACCCCGATAAGGACGAACAGCTGAGCAGGGGTTCTCAAGTCGCGGTGTCAACGGGCGGCTTTTTGAATGGAAACAGGTTTTTCAAT
GTGTATAACTTGAGAACGAGTAAGTAAATCATTATTGCGAATTTTATTTTATTGAAATATGCTAATATATTTTTTTGATCTTATATTTATTGGTA
TACTTGGCCGATGTCACTATTTATCAGAACTATTTCTCAGTAGAATTCATATATTTAACTTATAATTTTAAGAAGTAAACAACGAATTTTAAAT
CGTAGGATAACGAAGACTAAGAACATCGCCGTCTTAAATACCAAAACAAAAAAAAAACTATTTCTAAGTTACCTACTTACTTCAATAATATA
AATGTCAACTCTAATAACTTCATCCATTTACTTATCTTTTCTAAATTTGGAATATTTATTATCTAAAATTATAAATAAATATGAAACAAACCAC
ATTTTAAATCAGTAGTATAATAAAAAATACAATTGAAAAACATCAACAGCACGAATCTGCAACATTTGCAACCCAACAGTTGAGAAGCTCTG
TACTATACACAAACACAAACAACAGGTACAACAGCTGACTTGTGTTTGGTTCGAGAATTTTCTACTAGATACTAGATGGCGCTGTTGCCAAT
ATAAAAGGCGGCCGAAGCGCGGCACGGGCATCAGTTTTGAAAACAAGCGAACAAGTGATACAAACTTCAAGCGCTCTCAAAGCGACTCG
AGGATATTTCTCAGCGATTTTTGTGAAGGAAGAAAGTTGTTGTTGTGTTAGAAATCAAGCAAA

Figure S3. Promoter sequence of SfHsp20.71 gene with potential HSEs marked. The 1783 bp nucleic acid sequence with three types of HSE elements present upstream to the ATG of SfHsp20.71 gene are shown. Two types of HSEs, tail-tail and step/gap, indicated by red and blue bold lines respectively are shown on the top of the sequence. Another type of HSE, head-head, represented by pink bold lines, is displayed below the sequence.

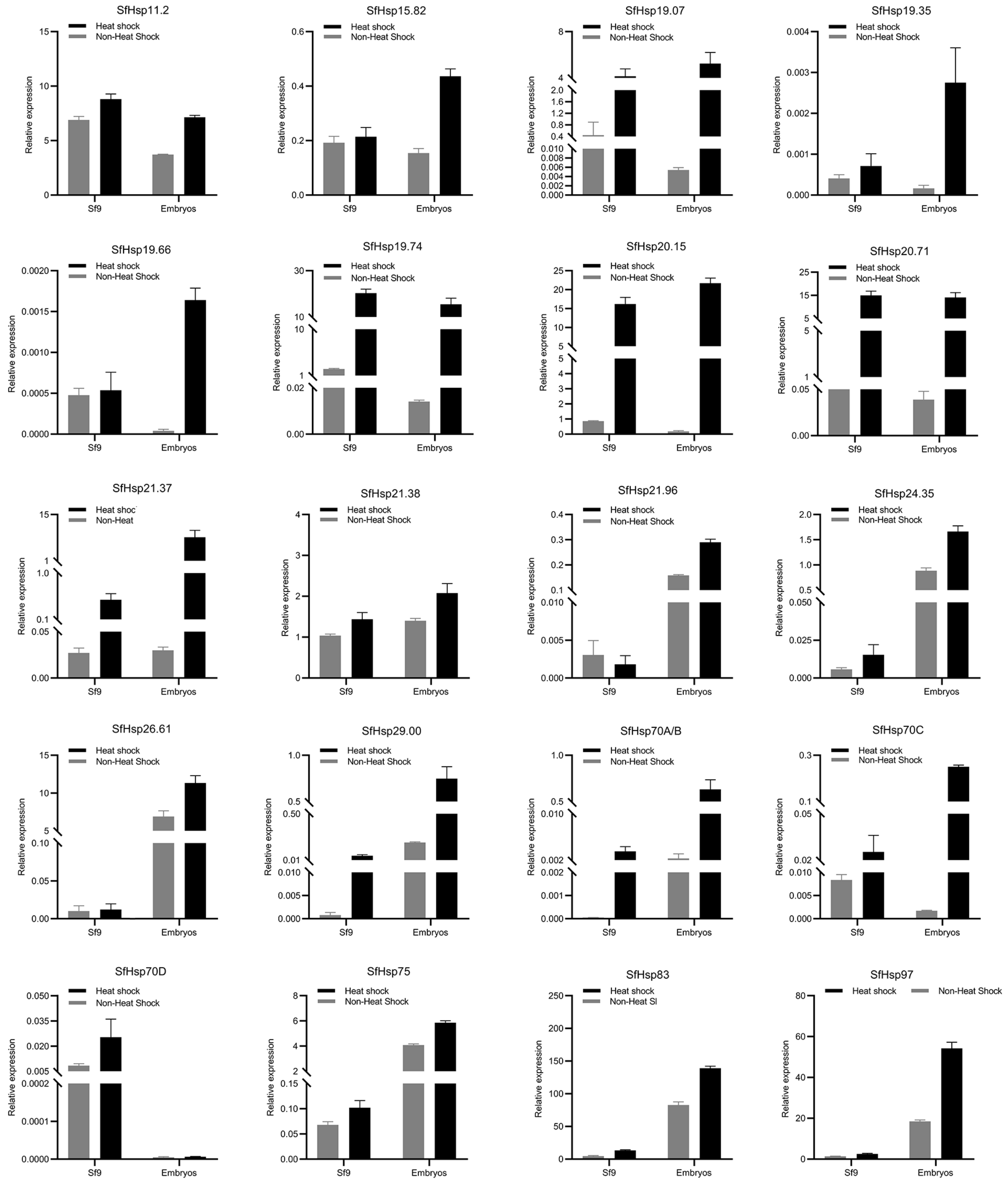

Figure S4. Relative mRNA levels of *SfHsp* genes in Sf9 cells and embryos. The Sf9 cells or embryos were exposed to 37℃ for 1 hr, then let them recover at 27℃ for 1 hr. Cells and fresh embryos kept at

27 ℃ were used as non-heat-shock control. Total RNA was isolated, converted to cDNA and used in RT-qPCR to quantify mRNA levels. 28 srRNA gene as the reference gene. Mean ± SD (n=3) are shown.

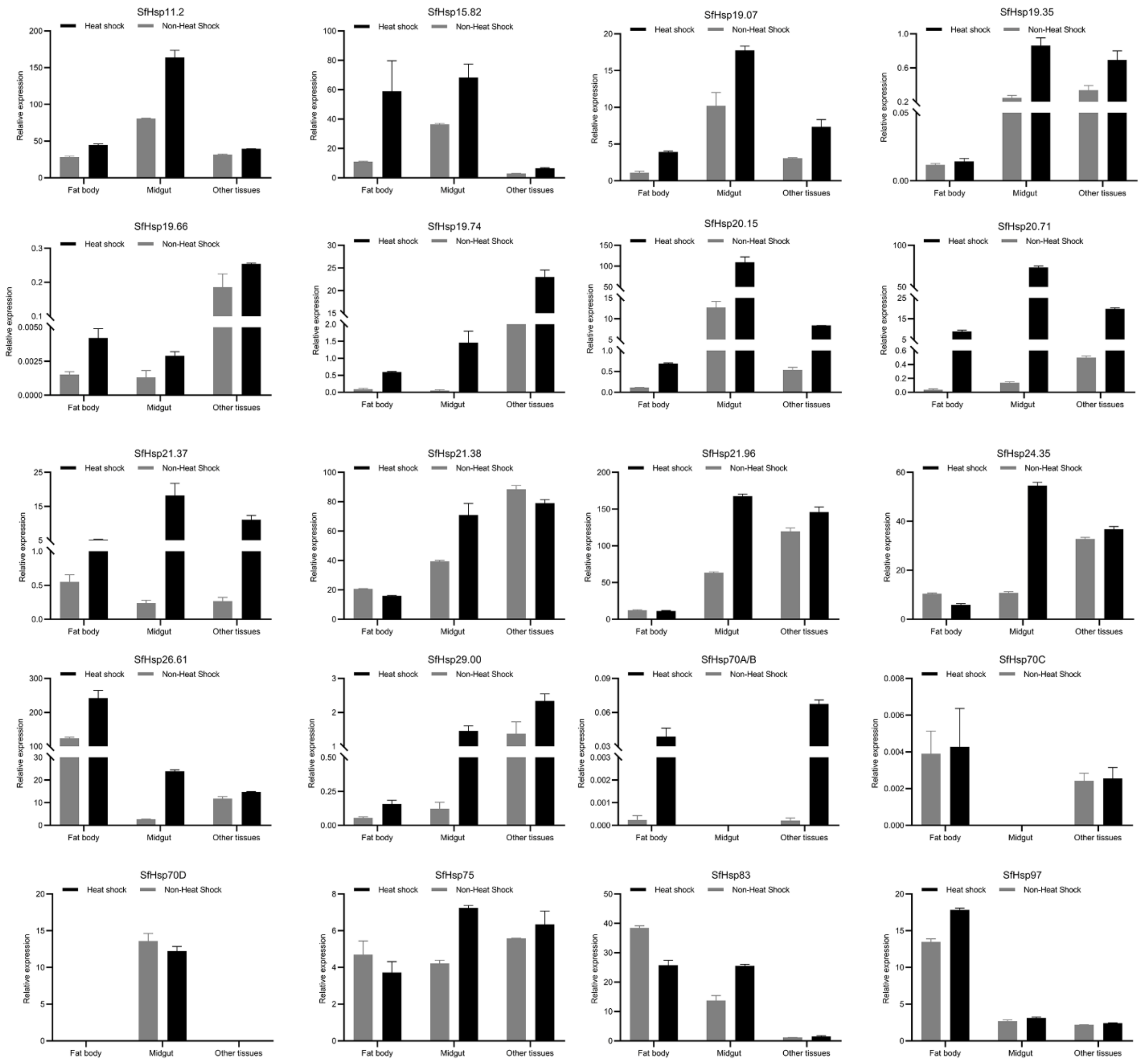

Figure S5. Relative mRNA expression levels of *SfHsp* genes in different larval tissues. The 6th instar larvae were exposed to 37 ℃ for 1 hr, then recovered at 27℃ for 1 hr. Fat body, midgut, and remaining tissues were dissected. Tissues dissected from larvae raised at 27℃ were used as non-heat-shock control. Mean ± SD (n=3) are shown.

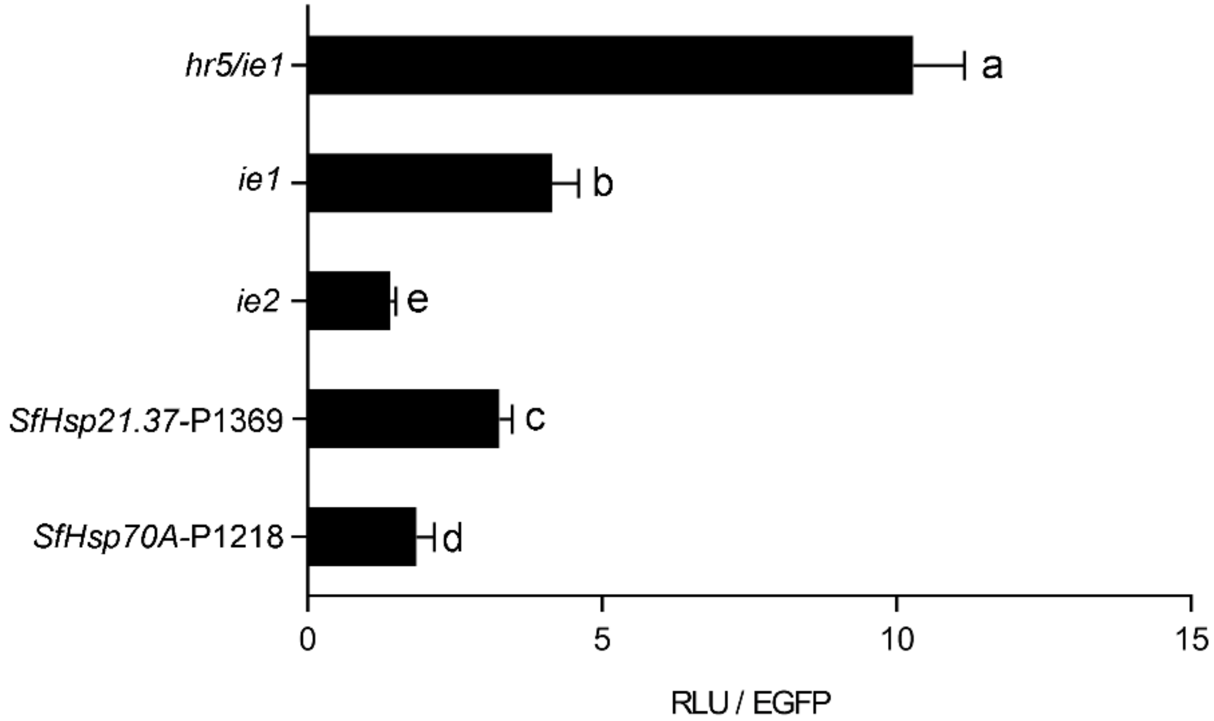

Figure S6. Comparison of highly heat-inducible *S. frugiperda* heat-shock promoters with commercially used promoters in Sf9 cells. Top two heat-shock promoter constructs that showed heat-inducible activity in Sf9 cells, and ie1, ie2, and hr5/ie1 constructs were co-transfected into Sf9 cells with a construct expressing EGFP. The transfected cells were processed as described in Figure 4. The fluorescence intensity of EGFP in each sample was determined using SpectraMax i3x with excitation maximum of 484 nm and emission maximum of 509 nm. The luciferase activity was normalized by dividing with the EGFP activity in the same sample. Mean ± SD (n=4) are shown. Different letters beside each column indicate significant differences ($p < 0.05$) among groups determined using One-way ANOVA followed by the Tukey HSD test.
